# Survival rate in patients with ICU-acquired infections and its related factors in Iran’s hospitals

**DOI:** 10.1186/s12889-021-10857-y

**Published:** 2021-04-24

**Authors:** MEDSKorosh Etemad, Yousef Khani, Seyed-Saeed Hashemi-Nazari, Neda Izadi, Babak Eshrati, Yadollah Mehrabi

**Affiliations:** 1 Department of Epidemiology, School of Public Health and Safety, Shahid Beheshti University of Medical Sciences, Tehran, Iran; 2 Clinical Research Development Unit, Shahid Madani Hospital, Alborz University of Medical Sciences, Karaj, Iran; 3 Prevention of Cardiovascular Disease Research Center, Department of Epidemiology, School of Public Health and Safety, Shahid Beheshti University of Medical Sciences, Tehran, Iran; 4 Department of Social Medicine, School of Medicine, Iran University of Medical Sciences, Tehran, Iran

**Keywords:** Hospital-acquired infections, Survival rate, Risk factors, Intensive care units, Iran

## Abstract

**Background:**

Hospital-acquired infections (HAIs) are a well-known cause of morbidity and mortality in hospitalized patients. This study aimed at investigating the survival rate in patients with ICU-acquired infections (ICU-AIs) and its related factors in Iran’s hospitals.

**Methods:**

Data were obtained from the Iranian Nosocomial Infections Surveillance (INIS), which registers all necessary information on the main types of infection from different units of each included hospital. One thousand one hundred thirty-four duplicate cases were removed from the analysis using the variables of name, father’s name, age, hospital code, infection code, and bedridden date. From 2016 to 2019, 32,998 patients diagnosed with ICU-AI from about 547 hospitals. All patients were followed up to February 29, 2020.

**Results:**

The median age of patients with ICU-AIs was 61 (IQR = 46) years. 45.5, 20.69, 17.63, 12.08, and 4.09% of infections were observed in general, surgical, internal, neonatal and pediatric ICUs, respectively. Acinetobacter (16.52%), E.coli (12.01%), and Klebsiella (9.93%) were the major types of microorganisms. From total, 40.76% of infected patients (13,449 patients) died. The 1, 3, 6-months and overall survival rate was 70, 25.72, 8.21 1.48% in ICU-AI patients, respectively. The overall survival rate was 5.12, 1.34, 0.0, 51.65, and 31.08% for surgical, general, internal, neonatal and pediatric ICU, respectively. Hazard ratio shows a significant relationship between age, hospitalization-infection length, infection type, and microorganism and risk of death in patients with ICU-AI.

**Conclusions:**

Based on the results, it seems that the nosocomial infections surveillance system should be more intelligent. This intelligence should act differently based on related factors such as the age of patients, hospitalization-infection length, infection type, microorganism and type of ward. In other words, this system should be able to dynamically provide the necessary and timely warnings based on the factors affecting the survival rate of infection due to the identification, intervention and measures to prevent the spread of HAIs based on a risk severity system.

**Supplementary Information:**

The online version contains supplementary material available at 10.1186/s12889-021-10857-y.

## Introduction

Hospital-acquired infections (HAIs) are infections that are not present in patients at the time of hospital admission and also are not in the incubation period. These infections occur after hospitalization, especially within 48–72 h after hospital admission and up to 6 weeks later [1].

These infections are a well-known cause of morbidity and mortality in hospitalized patients which in fact complicate hospitalization and treatment processes [2, 3].

These infections include the ventilator-associated events/ respiratory infections/pneumonia (VAE/VAP), central line-associated bloodstream infections/ septicemia (CLABSI), catheter-related urinary tract infection (CAUTI), surgical site infections (SSI), and clostridium difficile infections (CDI) [4]. In addition, the risk factors for these infections include the immunosuppression, older age, longer length of stay in hospital, multiple underlying comorbidities, frequent visits to healthcare facilities, mechanical ventilatory support, recent invasive procedures, indwelling devices, and stay in an intensive care unit (ICU) [5].

The risk of these infections is higher in the intensive care unit, so that in a study of 231,459 patients admitted to the intensive care unit of 947 hospitals in Germany (2015), the point prevalence of ICU-acquired infections (ICU-AIs) was 19.5% [6]. While in study by Magill et al. (2014) on 11,282 patients from 183 U.S. acute care hospitals, the overall point prevalence of these infections was only 4% [7].

Various studies have shown that the incidence of hospital-acquired infections in the intensive care unit is 5 to 10 times higher than in other hospital wards [8]. Some other studies have also shown that 50% of patients admitted to the intensive care unit affected from this infections [9, 10]. Accordingly, these infections are in fact, serious problem which complicate the patients in the intensive care unit [11]. Because the high prevalence of these infections in the intensive care unit, it leads to increase in morbidity and mortality of hospitalized patients. It also increase the economic burden due to higher prescription of antibiotics and longer hospital stay in hospitals [8, 11].

Although the prevalence of HAIs and its related factors have been widely studied, no special study has been conducted in Iran regarding their survival rate of such patients, especially those admitted in ICUs. Therefore, this study aimed at investigating the survival rate in patients with ICU-acquired infections and its related factors in Iran’s hospitals.

## Methods

In this retrospective cohort study, data were obtained from the Iranian Nosocomial Infections Surveillance (INIS), which uses the standard definitions provided by the national nosocomial infections surveillance system (NNIS). The NNIS registers four main types of infection, including the following: catheter-associated urinary tract infections (CAUTI), ventilator-associated events (VAE), surgical site infections (SSI), central line-associated bloodstream infections (CLABSI) and other infections (bone and joint, central nervous system, chorionic villus sampling, eye, ear, nose, throat and mouth, gastrointestinal system, reproductive tract infection, and skin and soft tissue infections). It also records different types of information in different units of each hospital [12]. For the purpose of this study, following variables were included: age, gender, hospitalization-infection length, duration of hospitalization, type of ward, type of infection, device use (different types of catheters include the urinary catheter, artery catheter, umbilical catheter, peripheral venous catheter, temporary central venous and permanent central venous catheter and also ventilator), type of microorganism, type of hospital, and death status. The primary outcome for this study was all cause death among patients with HAIs admitted to the ICU. Follow-up time was considered for one year, whether discharged or death.

### Statistical analysis

The median (Interquartile Range = IQR) and count (percentage) were used to describe quantitative and qualitative variables, respectively. The Chi-square test calculated the frequency of different variables among the two groups (alive and dead). The Kaplan Meier was used to compare survival rates between different groups. In addition, we used the Cox proportional hazard regression model to estimate the crude and adjusted hazard ratio (HR) for the risk of death among patients with ICU-acquired infection. To identify the factors related to survival in patients with ICU-acquired infections, all the variables with a *P*-value less than 0.2 or variables whose most levels are less than 0.2 at the univariable model were included in the multivariable analysis. Data were analyzed by the Stata (version 14.0; Stata Corp, Texas, USA) software. For all statistical tests *P* < 0.05 was considered statistically significant.

This study was approved by the ethical committee of National Institute for Medical Research Development (IR.NIMAD.REC.1399.074).

## Results

One thousand one hundred thirty-four duplicate cases were removed from the analysis by matching name, father’s name, age, hospital code, infection code, and bedridden date. Finally, the 32,998 patients diagnosed with ICU-AI from about 547 hospitals between 2016 and 2019 were eligible for analysis. The median age of 32,998 patients with ICU-acquired infections was 61 (IQR = 46) years. 58.28% (19,202) of patients were males. The median of hospitalization-infection length and hospitalization duration were 8 (IQR = 16) and 23 (IQR = 29) days, respectively. 45.5, 20.69, 17.63, 12.08, and 4.09% of infections were observed in general, surgical, internal, neonatal and pediatric ICUs, respectively. For 63.32% of patients, the device (catheter and ventilator) was used. The most common ICU-AIs were VAE (13,111 patients; 39.73%). The major type of microorganisms were Acinetobacter (16.52%), E.coli (12.01%), and Klebsiella (9.93%). From total, 40.76% of infected patients (13,449 patients) died. Variables associated with death of such patients were investigated and have been shown in Table 1.

**Table 1:** Factors associated with the all-cause mortality among ICU-acquired infections patients

Variable		Alive (19,549)	Dead (13,449)	Total (32,998)	*P*-value^*^
		N (%)	N (%)	N	
**Gender**	**Male**	11,782 (61.36)	7420 (38.64)	19,202	< 0.001
	**Female**	7727 (56.2)	6021 (43.8)	13,748	
**Hospitalization-infection length**	**≤8 days**	11,241 (65.09)	6030 (34.91)	17,271	< 0.001
	**> 8 days**	8308 (52.83)	7419 (47.17)	15,727	
**Ward type**	**Surgical ICU**	4087 (59.86)	2741 (40.14)	6828	< 0.001
	**General ICU**	8228 (54.8)	6786 (45.2)	15,014	
	**Internal ICU**	2743 (47.15)	3075 (52.85)	5818	
	**Pediatric ICU**	1030 (76.24)	321 (23.76)	1361	
	**Neonatal ICU**	3461 (86.81)	526 (13.19)	3987	
**Infection type**	**BSI**	2470 (58.45)	1756 (41.55)	4226	< 0.001
	**PENU & LRTI**	3404 (71.66)	1346 (28.34)	4750	
	**SSI**	1333 (71.13)	541 (28.87)	1874	
	**UTI**	4008 (61.66)	2492 (38.34)	6500	
	**VAE**	6421 (48.97)	6690 (51.03)	13,111	
	**Other**	1913 (75. 4)	624 (24. 6)	2537	
**Device use**	**Yes**	11,023 (52.76)	9870 (47.24)	20,893	< 0.001
	**No**	8526 (70.43)	3579 (29.57)	12,105	
**Device type**	**Catheters**	3633 (56.46)	2802 (43.54)	6435	< 0.001
	**Ventilators**	6820 (50.13)	6784 (49.87)	13,604	
	**Other**	570 (66.74)	284 (33.26)	854	
	**Without device**	8526 (70.43)	3579 (29.57)	12,105	
**Microorganism**	**Staphylococcus epidermidis**	885 (73.26)	323 (26.74)	1208	< 0.001
	**Staphylococcus aureus**	995 (62.66)	593 (37.34)	1588	
	**Coagulase negative staphylococci**	548 (66.26)	279 (33.74)	827	
	**Acinetobacter**	2617 (48.01)	2834 (51.99)	5451	
	**Escherichia coli**	2373 (59.89)	1589 (40.11)	3962	
	**Enterobacter**	901 (63.18)	525 (36.82)	1426	
	**Enterococcus**	435 (59.92)	291 (40.08)	726	
	**Pseudomonas aeruginosa**	1474 (58.28)	1055 (41.72)	2529	
	**Citrobacter**	328 (58.89)	229 (41.11)	557	
	**Candida**	467 (45.43)	561 (54.75)	1028	
	**Candida albicans**	284 (55.15)	231 (44.85)	515	
	**Klebsiella**	1875 (57.23)	1401 (42.77)	3276	
	**Klebsiella pneumoniae**	921 (48.78)	967 (51.22)	1888	
	**Other**	1804 (62.6)	1078 (37.4)	2882	
	**Unknown**	3642 (70.93)	1493 (29.07)	5135	
**Hospital type**	**Government**	16,121 (59.45)	10,998 (40.55)	27,119	< 0.001
	**Semi-government**	581 (43.78)	746 (56.22)	1327	
	**Private**	2045 (63.14)	1194 (36.86)	3239	
	**Other**	516 (53.86)	442 (46.99)	958	

*Based on Chi-square test

*BSI* Blood Stream Infections, *PENU & LRI* Pneumonia Events & Lower Respiratory Tract Infection, *SSI* Surgical Site Infection, *UTI* Urinary Tract Infection, *VAE* Ventilator Associated Events.

### The survival rate

**Fig. 1:**
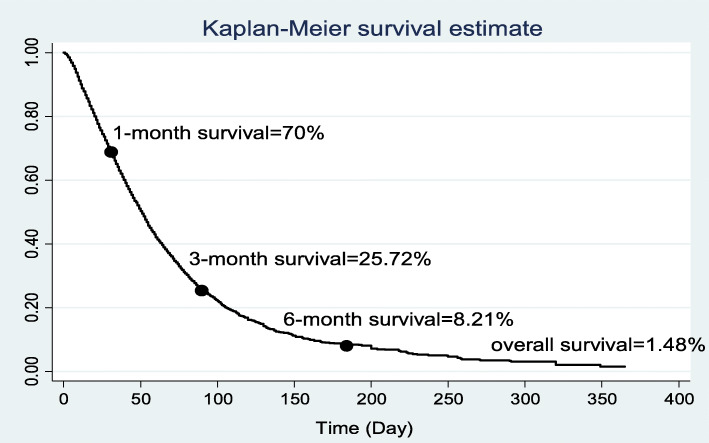
The 1, 3, 6-month, 1-year and overall survival in ICU-acquired infections patients

**Fig. 2:**
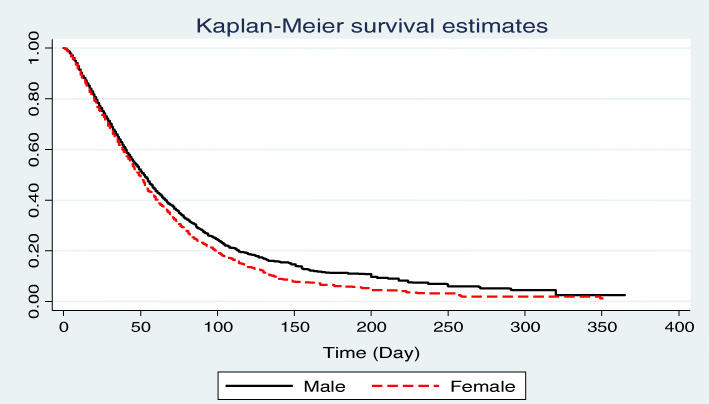
The overall survival among ICU-acquired infections patients by gender

**Fig. 3:**
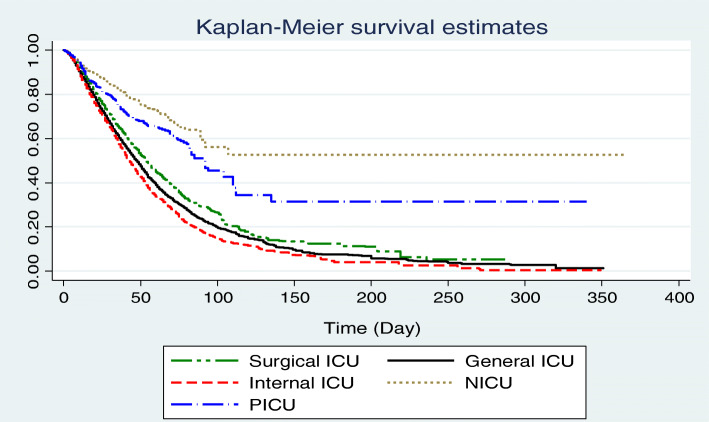
The overall survival among ICU-acquired infections patients by ward type

The 1, 3, 6-months and overall survival rate was 70, 25.72, 8.21 1.48% in ICU-acquired infections patients, respectively (Fig. 1). The 1, 3, 6-months and overall survival rates in males were higher than females (Fig. 2). The overall survival rate was 5.12, 1.34, 0.0, 51.65, and 31.08% for surgical, general, internal, neonatal and pediatric ICU, respectively. Also, the 6-months and the overall survival rate among neonates (51.65%) was significantly higher compared to other age groups (Fig. 3).

### The risk of death in patients with ICU-acquired infection

The association between different variables and risk of death were investigated and have been presented in Appendix 1, Table 2.

**Table 2:** Multiple Cox regression analysis of survival of ICU-acquired infection patients by ward type

Variable		Adjusted HR (95% CI)				
		General ICU	Internal ICU	Surgical ICU	Pediatric ICU	Neonatal ICU
**Age**	**Year**	1.01* (1.009–1.01)	1.009* (1.007–1.01)	1.017* (1.015–1.019)	0.99 (0.98–1)	–
**Gender**	**Male**	1	1	1	1	1
	**Female**	1.06* (1.01–1.11)	1.06 (0.99–1.14)	1.13* (1.05–1.22)	–	–
**Hospitalization-infection length**	**≤8 days**	1	1	1	1	1
	**> 8 days**	0.36* (0.34–0.38)	0.37* (0.34–0.4)	0.42* (0.39–0.46)	0.45* (0.35–0.57)	0.39* (0.32–0.48)
**Infection type**	**BSI**	–	1.5* (1.15–1.95)	1.5* (1.26–1.78)	–	2.35* (1.3–4.26)
	**PENU & LRI**	–	1.35* (1.02–1.77)	0.96 (0.79–1.17)	–	1.26 (0.69–2.31)
	**SSI**	1	1	1	1	1
	**UTI**	–	1.23 (0.95–1.59)	1.04 (0.87–1.24)	–	0.56 (0.27–1.18)
	**VAE**	–	1.51* (1.19–1.93)	1.33* (1.14–1.54)	–	2.05* (1.09–3.87)
	**Other**	–	1.43* (1.07–1.92)	1.9* (1.54–2.34)	–	0.62 (0.33–1.17)
**Device use**	**No**	1	1	1	1	1
	**Yes**	1.16* (1.1–1.23)	–	–	1.19 (0.95–1.48)	1.26* (1.02–1.56)
**Microorganism**	**Staphylococcus epidermidis**	1	1	1	1	1
	**Staphylococcus aureus**	0.98 (0.81–1.19)	1.31 (0.99–1.74)	1.56* (1.1–2.2)	–	0.52 (0.21–1.27)
	**Coagulase negative staphylococci**	1.1 (0.88–1.38)	1.54* (1.08–2.19)	1.5* (1.01–2.21)	–	0.76 (0.4–1.46)
	**Acinetobacter**	1.25* (1.06–1.48)	1.31* (1.03–1.66)	1.97* (1.45–2.68)	–	2.91* (1.86–4.54)
	**Escherichia coli**	1.09 (0.92–1.3)	1.41* (1.1–1.82)	1.77* (1.29–2.42)	–	1.41 (0.83–2.39)
	**Enterobacter**	1.04 (0.85–1.27)	1.27 (0.96–1.69)	1.32 (0.91–1.9)	–	2.92* (1.77–4.81)
	**Enterococcus**	0.97 (0.77–1.23)	1.26 (0.9–1.76)	2.09* (1.44–3.03)	–	1.74 (0.84–3.61)
	**Pseudomonas aeruginosa**	0.9 (0.75–1.08)	1.02 (0.78–1.33)	1.41* (1.01–1.95)	–	2.13* (1.21–3.75)
	**Citrobacter**	1.05 (0.73–1.32)	1.19 (0.75–1.88)	1.49* (1.01–2.2)	–	2.25 (0.97–5.17)
	**Candida**	1.23* (1.01–1.51)	1.54* (1.15–2.06)	2.76* (1.95–3.9)	–	4.9* (2.22–10.81)
	**Candida albicans**	1.26 (0.99–1.61)	1.42 (0.99–2.05)	1.64* (1.09–2.47)	–	2.95* (1.33–6.5)
	**Klebsiella**	0.97 (0.82–1.16)	1.4* (1.09–1.8)	1.62* (1.17–2.24)	–	2.52* (1.56–4.04)
	**Klebsiella pneumoniae**	0.96 (0.8–1.15)	1.29 (0.98–1.69)	1.77* (1.29–2.45)	–	2.3* (1.3–4.08)
	**Other**	0.99 (0.83–1.19)	1.16 (0.89–1.52)	1.61* (1.36–2.56)	–	1.56 (0.96–2.55)
	**Unknown**	1.25* (1.05–1.49)	1.4* (1.08–1.81)	1.87* (1.17–2.22)	–	1.86* (1.19–2.9)
**Hospital type**	**Government**	–	1.34* (1.09–1.64)	–	–	–
	**Semi-government**	–	1.5* (1.16–1.92)	–	–	–
	**Private**	1	1	1	1	1
	**Other**	–	1.07 (0.84–1.37)	–	–	–

*P < 0.05

*HR* Hazard Ratio, *CI* Confidence Interval, *BSI* Blood Stream Infections, *PENU & LRI* Pneumonia Events & Lower Respiratory Tract Infection, *SSI* Surgical Site Infection, *UTI* Urinary Tract Infection, *VAE* Ventilator Associated Events.

#### General ICU

Based on multivariable Cox regression, with increasing one year of age, the risk of death in ICU-AI patients increases by 1% (HR = 1.01; 95% CI: 1.009–1.01). The risk of death was 6% higher in women than men (HR = 1.06; 95% CI: 1.01–1.011). The hospitalization-infection length > 8 days was associated with a 64% decrease in the number of deaths in ICU-acquired infections (HR = 0.36; 95% CI: 0.34–0.38). The use of a device (catheter and ventilator) was also associated with a 16% increase in death risk (HR = 1.16; 95% CI: 1.1–1.23). Considering the types of microorganisms, the largest increase in death risk was related to the Acinetobacter (HR = 1.25; 95% CI: 1.06–1.48) (Table 2).

#### Internal ICU

The risk of death in ICU-AI patients increases by 0.9% (HR = 1.009; 95% CI: 1.007–1.01) with increasing 1 year of age. The hospitalization-infection length > 8 days was associated with a 63% decrease in the number of death in ICU-acquired infections patients (HR = 0.37; 95% CI: 0.34–0.4). In addition, the risk of death was 34% (HR = 1.34; 95% CI: 1.09–1.64) and 50% (HR = 1.5; 95% CI: 1.16–1.92) higher in government and semi-government hospitals compare to private hospitals. Considering the types of microorganisms, the largest increase in the risk of death was related to the Candida (HR = 2.76; 95% CI: 1.95–3.9), Enterococcus (HR = 2.09; 95% CI: 1.44–3.03), and Acinetobacter (HR = 1.97; 95% CI: 1.45–2.68) (Table 2).

#### Surgical ICU

With increasing 1 year of age, the risk of death in ICU-AI patients increases by 1.7% (HR = 1.017; 95% CI: 1.015–1.019). The risk of death was 13% higher in women than men (HR = 1.13; 95% CI: 1.05–1.22). The hospitalization-infection length > 8 days was associated with a 58% decrease in the number of deaths in ICU-acquired infections (HR = 0.42; 95% CI: 0.39–0.46). In addition, based on type of infection, BSI, VAE and other type of infections increase the risk of death by 50% (HR = 1.5; 95% CI: 1.26–1.78), 33% (HR = 1.33; 95% CI: 1.14–1.54), and 90% (HR = 1.9; 95% CI: 1.54–2.34), respectively. Considering the types of microorganisms, the largest increase in the risk of death was related to the Candida (HR = 1.54; 95% CI: 1.15–2.06), Coagulase negative staphylococci (HR = 1.54; 95% CI: 1.08–2.19), and Escherichia coli (HR = 1.41; 95% CI: 1.1–1.82) (Table 2).

#### Pediatric ICU

The risk of death in PICU was only associated with hospitalization-infection length (HR = 0.45; 95% CI: 0.35–0.57) (Table 2).

#### Neonatal ICU

The hospitalization-infection length > 8 days was associated with a 61% decrease in the number of death in ICU-acquired infections patients (HR = 0.39; 95% CI: 0.32–0.48). Based on the type of infection, BSI and VAE increase death risk (HR = 2.35; 95% CI: 1.3–4.26) and (HR = 2.05; 95% CI: 1.09–3.87). In addition, the use of a device (catheter and ventilator) was also associated with a 26% increase in the risk of death (HR = 1.26; 95% CI: 1.02–1.56). Considering the types of microorganisms, the largest increase in the risk of death was related to the Candida (HR = 4.9; 95% CI: 2.22–10.81), Candida Albicans (HR = 2.95; 95% CI: 1.33–6.5), Enterobacter (HR = 2.92; 95% CI: 1.77–4.81), and Acinetobacter (HR = 2.91; 95% CI: 1.86–4.54) (Table 2).

## Discussion

Our study showed that the survival rate in HAIs patients decreases over time. The reason for such decrease is multifactorial and is at least partly due to the possible change of pattern of infection over time, in which more than one organism may contribute to occurrence of HAIs. This in turn might be due to improper treatment of HAIs, multi-drug resistance, low quality of care for patients after their discharge from hospital [13–16].

According to the results of this study, the risk of death in women with HAIs admitted to the intensive care unit in general and surgical ICUs was higher than men. This result needs further investigation because the results from elsewhere is not consistent [8, 17]. However, the results of our study seem to be more reliable considering that its sample size is larger than most of other reports. Our results, are in fact, based on a national registration system.

In terms of type of ICU, the survival rate was higher in neonatal and pediatric ICUs and it may be because of the effect of age which is the main variable differed among such patients. Our finding is consistent with reports from elsewhere [18–21].

Studies have shown that increasing the length of hospitalization in the ICUs, increases the risk of HAIs. However, they do not necessarily increase the risk of death [22–25]. The main finding of this study was that hospitalization in the ICU for more than 8 days increases the survival in patients with nosocomial infections. The reason for such improvement in survival might be due to better care given to such patients which in turn lead to have a better condition for them [13]. Of course, it’s worthwhile to mention that the overall survival rate decreases with the prolong hospitalization in ICUs which is different based on type of microorganism.

Based on our study, blood stream infections (BSI) and infections associated with ventilator and catheter events lead to an increased risk of death and decreased survival rate in patients with HAIs admitted to the ICU. In our study, the most common microorganisms that increased the risk of death and decreased survival rate were: Candida Albicans, Candida, Acinetobacter, Enterococcus and Enterobacter. These findings are consistent with the results of other reports [13, 26–29]. Therefore, it is of high importance to pay special attention to these microorganisms, while trying to prevent their occurrence, considering appropriate and adequate treatment for each of these cases are essential.

This study was the first comprehensive and national study conducted on the survival rate in patients with ICU-acquired infections and its related factors in Iran’s hospitals using a nationwide registered data. Although, its coverage is about 83.5% and therefore it is not generalizable to not cover hospitals, but a random selection of all included hospitals almost represent the national situation of ICU-AIs and provide a national view.

On the other hand, the surveillance data are often limited in terms of recorded variables. Different scoring systems based on clinical and laboratory findings that predict the mortality in patients with critical situations such as the acute physiology and chronic health evaluation (APACHE) score, sequential organ failure assessment (SOFA), mortality in emergency department sepsis (MEDS), and other similar scoring systems are not covered in the INIS dataset. Therefore, we were not able to assess the effect of such variables on mortality at ICU admission.

## Conclusions

Despite the above mentioned limitations of the nosocomial infection surveillance system, the results of our report, provided a general picture for the survival rate and its related risk factors among ICU-AIs. It seems that the nosocomial infections surveillance system should be more intelligent. This intelligence should act differently based on related factors such as the age of patients, hospitalization-infection length, infection type, microorganism and type of ward. In other words, this system should be able to dynamically provide the necessary and timely warnings based on the factors affecting the survival rate of infection due to the identification, intervention and measures to prevent the spread of HAIs based on a risk severity system.

## Supplementary Information

**Additional file 1: Appendix 1.** Univariable Cox regression analysis of survival of ICU-acquired infection patients by ward.

## Data Availability

The datasets used and/or analysed during the current study are available from the corresponding author on reasonable request.

## Abbreviations

CAUTI: Catheter-related urinary tract infection
CLABSI: Central line-associated bloodstream infections / septicemia
HAIs: Hospital-acquired infections
ICU-AIs: ICU-acquired infections
ICUs: Intensive care units
INIS: Iranian nosocomial infections surveillance
IQR: Interquartile range
VAE/VAP: Ventilator-associated events / respiratory infections / pneumonia
HR: Hazard ratio
CI: Confidence interval
SSI: Surgical site infections
CDI: Clostridium difficile infections
APACHE: Acute physiology and chronic health evaluation
SOFA: Sequential organ failure assessment
MEDS: Mortality in emergency department sepsis
NNIS: National nosocomial infections surveillance system
PENU & LRI: Pneumonia events & lower respiratory tract infection

## References

[1] Horan TC, Andrus M, Dudeck MA (2008). CDC/NHSN surveillance definition of health care-associated infection and criteria for specific types of infections in the acute care setting. Am J Infect ControlAm J Infect Control..

[2] Higuera F, Rangel-Frasusto M, Rosenthal V, Soto J, Castanon J, Franco G (2007). Attributable cost and length of stay for patients with central venous catheter-associated bloodstream infection in Mexico City intensive care units: a prospective, matched analysis. Infect Control Hosp Epidemiol.

[3] Rosenthal VD, Guzman S, Migone O, Crnich CJ (2003). The attributable cost, length of hospital stay, and mortality of central line-associated bloodstream infection in intensive care departments in Argentina: a prospective, matched analysis. Am J Infect Control.

[4] Boev C, Kiss E (2017). Hospital-acquired infections: current trends and prevention. Critical Care Nurs Clin.

[5] Sydnor ER, Perl TM (2011). Hospital epidemiology and infection control in acute-care settings. Clin Microbiol Rev.

[6] Stiller A, Schröder C, Gropmann A, Schwab F, Behnke M, Geffers C, et al. ICU ward design and nosocomial infection rates: a cross-sectional study in Germany. J Hosp Infect. 2017;95(1):71–5. 10.1016/j.jhin.2016.10.011. 10.1016/j.jhin.2016.10.011 27884473

[7] Magill SS, Edwards JR, Bamberg W, Beldavs ZG, Dumyati G, Kainer MA, et al. Multistate point-prevalence survey of health care–associated infections. N Engl J Med. 2014;370(13):1198–208. 10.1056/NEJMoa1306801. 10.1056/NEJMoa1306801 PMC4648343 24670166

[8] Rejeb MB, Sahli J, Chebil D, Khefacha-Aissa S, Jaidane N, Kacem B (2016). Mortality among patients with nosocomial infections in tertiary Intensive Care Units of Sahloul hospital, Sousse, Tunisia. Arch Iran Med.

[9] Tomaszewski D, Rybicki Z, Duszyńska W (2019). The polish prevalence of infection in intensive care (PPIC): a one-day point prevalence multicenter study. Advanc Clin Exp Med.

[10] Vincent J, Rello J, Marshall J, Silva E, Anzueto A, Martin C, et al. International study of the prevalence and outcomes of infection in intensive care units. JAMA. 2009;302:2323–9. 10.1001/jama.2009.1754 19952319

[11] Singer M, Webb A (2009). Oxford handbook of critical care: OUP Oxford.

[12] Izadi N, Eshrati B, Etemad K, Mehrabi Y, Hashemi-Nazari S-S. Rate of the incidence of hospital-acquired infections (HAIs) in Iran based on the data of the national nosocomial infections surveillance. New Microbes New Infections. 2020;100768. 10.1016/j.nmni.2020.100768 PMC7568181 33093962

[13] Soneja M, Khanna P. Infectious Diseases in the Intensive Care Unit. Springer Nature Singapore Pte Ltd; 2020. 10.1007/978-981-15-4039-4_12.

[14] Despotovic A, Milosevic B, Milosevic I, Mitrovic N, Cirkovic A, Jovanovic S, et al. Hospital-acquired infections in the adult intensive care unit—epidemiology, antimicrobial resistance patterns, and risk factors for acquisition and mortality. Am J Infect Control. 2020;48(10):1211–5. 10.1016/j.ajic.2020.01.009. 10.1016/j.ajic.2020.01.009 32093978

[15] Schwab F, Geffers C, Behnke M, Gastmeier P (2018). ICU mortality following ICU-acquired primary bloodstream infections according to the type of pathogen: a prospective cohort study in 937 Germany ICUs (2006-2015). PLoS One.

[16] de la Varga-Martínez O, Gómez-Sánchez E, Muñoz MF, Lorenzo M, Gómez-Pesquera E, Poves-Álvarez R (2020). Impact of nosocomial infections on patient mortality following cardiac surgery. J Clin Anesth.

[17] Habibi S, Wig N, Agarwal S, Sharma SK, Lodha R, Pandey RM, et al. Epidemiology of nosocomial infections in medicine intensive care unit at a tertiary care hospital in northern India. Trop Dr. 2008;38(4):233–5. 10.1258/td.2008.070395. 10.1258/td.2008.070395 18820195

[18] Ebrahimzadeh A, Allahyari E, Nikoomanesh F, Zare Bidaki M (2020). A Comparative Analysis of Nosocomial Infections between Internal and Surgical Intensive Care Units of a University Hospital in Birjand, Iran from 2015 to 2016: A Retrospective Study.

[19] Zingg W, Holmes A, Dettenkofer M, Goetting T, Secci F, Clack L, et al. Hospital organisation, management, and structure for prevention of health-care-associated infection: a systematic review and expert consensus. Lancet Infect Dis. 2015;15(2):212–24. 10.1016/S1473-3099(14)70854-0. 10.1016/S1473-3099(14)70854-0 25467650

[20] Custovic A, Smajlovic J, Hadzic S, Ahmetagic S, Tihic N, Hadzagic H (2014). Epidemiological surveillance of bacterial nosocomial infections in the surgical intensive care unit. Materia socio-medica.

[21] Moreira MR, Guimarães MP, AADA R, Gontijo Filho PP (2013). Antimicrobial use, incidence, etiology and resistance patterns in bacteria causing ventilator-associated pneumonia in a clinical-surgical intensive care unit. Rev Soc Bras Med Trop.

[22] Khazaei S, Ayubi E, Jenabi E, Bashirian S, Shojaeian M, Tapak L. Factors associated with in-hospital death in patients with nosocomial infections: a registry base study according community data in west of Iran. Epidemiol Health. 2020;42:e2020037. 10.4178/epih.e2020037. 10.4178/epih.e2020037 PMC7644946 32512662

[23] Talaat M, Hafez S, Saied T, Elfeky R, El-Shoubary W, Pimentel G (2010). Surveillance of catheter-associated urinary tract infection in 4 intensive care units at Alexandria university hospitals in Egypt. Am J Infect Control.

[24] Ding J-G, Sun Q-F, Li K-C, Zheng M-H, Miao X-H, Ni W (2009). Retrospective analysis of nosocomial infections in the intensive care unit of a tertiary hospital in China during 2003 and 2007. BMC Infect Dis.

[25] Dhaneria M, Jain S, Singh P, Mathur A, Lundborg CS, Pathak A (2018). Incidence and determinants of health care-associated blood stream infection at a neonatal intensive care unit in Ujjain, India: a prospective cohort study. Diseases..

[26] Reddy PS, John MS, Devi PV, Kumar SS (2016). Nosocomial infections among patients admitted in general ICU: study from a tertiary-care hospital in South India. Int J Med Sci Pub Health.

[27] Ašembergienė J, Gurskis V, Kėvalas R, Valintėlienė R (2009). Nosocomial infections in the pediatric intensive care units in Lithuania. Medicina..

[28] Khazaei S, Adabi M, Bashirian S, Shojaeian M, Bathaei SJ, Karami M. Epidemiological profile of nosocomial infections among pediatric patients in a referral hospital in Hamadan, west of Iran. New Microbes and New Infections. 2020;100823. 10.1016/j.nmni.2020.100823 PMC7750152 33364030

[29] Comas-García A, Aguilera-Martínez JI, Escalante-Padrón FJ, Lima-Rogel V, Gutierrez-Mendoza LM, Noyola DE. Clinical impact and direct costs of nosocomial respiratory syncytial virus infections in the neonatal intensive care unit. Am J Infect Control. 2020;48(9):982–6 10.1016/j.ajic.2020.04.009 32305431

