# Supplementary material for: Survival rate in patients with ICU-acquired infections and its related factors in Iran’s hospitals
Source: BMC Public Health. 2021 Apr 24;21:787. doi: 10.1186/s12889-021-10857-y (PMC8065317; doi:10.1186/s12889-021-10857-y)
Supplement: Supplementary file 1 — Additional file 1: Appendix 1. Univariable Cox regression analysis of survival of ICU-acquired infection patients by ward. [file 12889_2021_10857_MOESM1_ESM.docx]

**Appendix 1.**

**Univariable Cox regression analysis of survival of ICU-acquired infection patients by ward type**

| **Variable** | | **Crude** **HR (95% CI)** | | | | |
| --- | --- | --- | --- | --- | --- | --- |
|  |  | **General ICU** | **Internal ICU** | **Surgical ICU** | **Pediatric ICU** | **Neonatal ICU** |
| **Age** | **Year** | 1.001*  (1.008-1.01) | 1.01*  (1.008-1.01) | 1.015*  (1.013-1.017) | 0.99  (0.98-1) | 0.99  (0.99-1.001) |
| **Gender** | **Male** | 1 | 1 | 1 | 1 | 1 |
|  | **Female** | 1.06*  (1.01-1.12) | 1.06  (0.99-1.14) | 1.21*  (1.12-1.31) | 1.14  (0.91-1.42) | 1.09  (0.92-1.3) |
| **Hospitalization-infection length** | **≤8 days** | 1 | 1 | 1 | 1 | 1 |
|  | **>8 days** | 0.37*  (0.35-0.39) | 0.37*  (0.34-0.4) | 0.46*  (0.43-0.50) | 0.45*  (0.35-0.57) | 0.43*  (0.36-0.52) |
| **Infection type** | **BSI** | 1.13  (0.97-1.3) | 1.45*  (1.12-1.89) | 1.38*  (1.17-1.64) | 1.54  (0.83-2.86) | 2.14*  (1.19-3.84) |
|  | **PENU & LRTI** | 1.2*  (1.03-1.39) | 1.7*  (1.3-2.23) | 1.04  (0.86-1.26) | 1.64  (0.89-2.99) | 1.47  (0.81-2.66) |
|  | **SSI** | 1 | 1 | 1 | 1 | 1 |
|  | **UTI** | 1.02  (0.89-1.17) | 1.34  (1.05-1.72) | 1.16  (0.98-1.36) | 1.85  (0.98-3.47) | 0.53  (0.25-1.1) |
|  | **VAE** | 1.3*  (1.14-1.48) | 1.64*  (1.29-2.08) | 1.41*  (1.22-1.64) | 1.39  (0.74-2.61) | 2.55*  (1.39-4.67) |
|  | **Other** | 1.1  (0.92-1.33) | 1.41*  (1.05-1.89) | 2.01*  (1.64-2.46) | 1.89  (0.98-3.62) | 0.6  (0.32-1.13) |
| **Device use** | **No** | 1 | 1 | 1 | 1 | 1 |
|  | **Yes** | 1.06*  (1.002-1.12) | 0.91  (0.81-1.01) | 1  (0.92-1.08) | 1.17  (0.94-1.46) | 1.98*  (1.66-2.36) |
| **Microorganism** | **Staphylococcus epidermidis** | 1 | 1 | 1 | 1 | 1 |
|  | **Staphylococcus aureus** | 1.07  (0.89-1.3) | 1.46*  (1.11-1.92) | 1.55*  (1.1-2.17) | 0.74  (0.27-1.98) | 0.47  (0.19-1.12) |
|  | **Coagulase negative staphylococci** | 1.22  (0.97-1.53) | 1.71*  (1.21-2.43) | 1.37  (0.93-2.03) | 1.08  (0.38-3.05) | 0.77  (0.4-1.47) |
|  | **Acinetobacter** | 1.29*  (1.09-1.52) | 1.21  (0.96-1.52) | 1.66*  (1.22-2.24) | 1.3  (0.64-2.67) | 2.54*  (1.64-3.92) |
|  | **Escherichia coli** | 1.24*  (1.05-1.48) | 1.19  (0.94-1.52) | 1.57*  (1.16-2.14) | 1.42  (0.66-3.04) | 0.94  (0.55-1.59) |
|  | **Enterobacter** | 1.005  (0.82-1.22) | 1.01  (0.77-1.34) | 1.09  (0.76-1.56) | 1.33  (0.59-3.02) | 1.94*  (1.19-3.18) |
|  | **Enterococcus** | 0.98  (0.78-1.24) | 1.02  (0.73-1.41) | 1.76*  (1.22-2.54) | 0.73  (0.25-2.05) | 1.08  (0.52-2.23) |
|  | **Pseudomonas aeruginosa** | 0.83*  (0.69-0.99) | 0.77  (0.6-1.001) | 1.08  (0.79-1.49) | 0.85  (0.4-1.78) | 1.71  (0.97-2.99) |
|  | **Citrobacter** | 1.06  (0.84-1.34) | 0.92  (0.59-1.44) | 1.38  (0.94-2.03) | 1.08  (0.29-4.008) | 1.28  (0.56-2.94) |
|  | **Candida** | 1.3*  (1.06-1.59) | 1.26  (0.96-1.65) | 1.98*  (1.42-2.76) | 1.62  (0.73-3.56) | 3.27*  (1.48-7.18) |
|  | **Candida albicans** | 1.38*  (1.08-1.75) | 1.08  (0.76-1.54) | 1.38  (0.92-2.06) | 1.12  (0.37-3.35) | 2.3*  (1.04-5.05) |
|  | **Klebsiella** | 0.99  (0.83-1.17) | 1.18*  (1.01-1.35) | 1.44*  (1.05-1.98) | 0.84  (0.37-1.89) | 1.65*  (1.03-2.63) |
|  | **Klebsiella pneumoniae** | 0.98  (0.82-1.18) | 0.99  (0.76-1.3) | 1.49*  (1.08-2.04) | 0.66  (0.24-1.79) | 1.35  (0.77-2.36) |
|  | **Other** | 1.04  (0.87-1.24) | 1.03  (0.79-1.34) | 1.36  (0.99-1.87) | 0.94  (0.46-1.92) | 1.31  (0.8-2.12) |
|  | **Unknown** | 1.37*  (1.14-1.63) | 1.47*  (1.15-1.87) | 1.96*  (1.44-2.67) | 1.19  (0.59-2.4) | 1.27  (0.85-1.9) |
| **Hospital type** | **Government** | 0.86  (0.71-1.01) | 1.33*  (1.09-1.63) | 1.13  (0.93-1.38) | 2.83  (0.39-20.18) | 1.17  (0.48-2.83) |
|  | **Semi-government** | 0.89  (0.79-1.001) | 1.27*  (1.0009-1.63) | 2.3*  (1.79-2.97) | omitted | 0.5  (0.15-1.67) |
|  | **Private** | 1 | 1 | 1 | 1 | 1 |
|  | **Other** | 0.76*  (0.64-0.89) | 0.92  (0.72-1.17) | 1.3  (0.95-1.78) | omitted | 1.33  (0.42-4.19) |

**HR=** Hazard Ratio; **CI=** Confidence Interval; *****P<0.05

**BSI=**Blood Stream Infections; **PENU & LRTI=**Pneumonia Events & Lower Respiratory Tract Infection; **SSI=**Surgical Site Infection; **UTI=**Urinary Tract Infection; **VAE=**Ventilator Associated Events
